# TLR7 Deficiency Leads to TLR8 Compensative Regulation of Immune Response against JEV in Mice

**DOI:** 10.3389/fimmu.2017.00160

**Published:** 2017-02-20

**Authors:** Muhammad Awais, Ke Wang, Xianwu Lin, Wenjie Qian, Nan Zhang, Chong Wang, Kunlun Wang, Ling Zhao, Zhen F. Fu, Min Cui

**Affiliations:** ^1^State Key Laboratory of Agricultural Microbiology, College of Veterinary Medicine, Huazhong Agricultural University, Wuhan, China; ^2^Department of Pathology, College of Veterinary Medicine, University of Georgia, Athens, GA, USA

**Keywords:** Japanese encephalitis virus, TLR7, TLR8, immune response, viral load

## Abstract

Japanese encephalitis virus (JEV) is a highly fatal pathogen to human beings. Toll-like receptor 7 (TLR7) plays a role as the first host defense against most single-stranded RNA flaviviruses. This study aims to investigate the role of TLR7 in inducing adaptive immune response in mice against JEV. *In vitro* and *in vivo* studies were conducted to examine the expression of toll-like receptors (TLRs) in mice. After JEV infection, physical parameters of mice (survival rate and body weight) were evaluated, and organs or cells were collected for further analysis. The expression of TLR7 was increased significantly as compare to other TLR molecules post-JEV infection. The expression of CD80, CD86, and CD273 on bone marrow-derived dendritic cells was increased significantly in TLR7^−/−^ mice. Furthermore, viral load was also increased significantly in TLR7^−/−^ mice as compare to C57BL/6 mice. But there was no significant difference among survival rate and body weight in TLR7^−/−^ mice as compare to C57BL/6. Interestingly, we also found that TLR8 was upregulated in TLR7^−/−^ mice. The study concluded that TLR8 was upregulated in TLR7-deficient mice, and it might play a compensatory role in the immune response in TLR7^−/−^ mice.

## Introduction

Japanese encephalitis virus (JEV), a single-stranded RNA (ssRNA) virus, belongs to the family Flaviviridae that causes approximately 50,000 cases of encephalitis each year in human (1). Although JEV is endemic in many parts of the world, its prevalence is high in under-developed countries of the Asian subcontinent (2). The transmission dynamics and pathogenesis of JEV have been widely studied (3, 4); however, the molecular mechanism involved in Japanese encephalitis (JE) still requires detailed investigation. JE is transmitted by infected mosquitoes and affects the central nervous system, resulting in encephalitis, meningitis, and poliomyelitis-like acute flaccid paralysis (5). JEV first amplify in periphery of the host in macrophages and dendritic cells (DCs) and, then, get entry into the CNS *via* the blood–brain barrier (BBB) (5). Within the CNS, JEV causes infection and directly kills the neurons (6). Viral replication within astrocytes and microglia cells has also been reported, which produces pro-inflammatory cytokines that cause indirect neuronal death (7).

Japanese encephalitis virus-specific T cells and virus neutralizing IgG and IgM antibodies perform a vital part in clearing the virus from CNS and peripheral lymphoid tissues (8). Emerging evidence also indicates that innate immune responses also play a crucial part for initial control of JEV infection and may also contribute to the inflammatory process (9, 10). Following viral infection, neurons and other CNS cells actively respond and produce type I interferon (IFN). The infection of West Nile Virus, another member of Flaviviridae family, also triggers the production of type 1 IFN (11). The virus prediction is done through MDA5 and RIG-I by pathogen-associated molecular patterns, which function as host germ-line pattern recognition receptors (PRRs) (12). Ablation of these PRRs and their downstream signaling molecules (MAVS) or transcriptional activators interferon regulatory transcription factor (IRF-3 and IRF-7) results in greatly enhanced susceptibility to WNV infection (13). There is also accumulating evidence that the type 1 IFN pathway may be activated through the recognition of toll-like receptors (TLRs) (14, 15). RIG-I and MDA5 recognize RNA or ssRNA in the cytosol and subsequently transmit signals through the adaptor molecule MAVS. Surface bound or endosomal TLRs recognize both ssRNA and dsRNA and signal through Myd88 and TRIF molecules (16, 17).

Neurotropic flaviviruses trigger innate immune responses in both a MyD88-dependent and MyD88-independent manner, suggesting the involvement of TLR signaling in flaviviral-mediated immune responses (18). A recent study of TLR-deficient mice indicated that TLR4 and TLR3 have distinct signaling functions after JEV infection. TLR3-deficient mice were highly susceptible to JE. TLR3^−/−^ mice exhibited increased pro-inflammatory cytokines and increased BBB permeability. Significantly enhanced viral loads were also observed in TLR3-deficient mice. TLR4-deficient mice demonstrated enhanced resistance to JEV infection. TLR4 deficiency induced potent type I IFN responses through enhanced stimulation of antiviral ISG genes by different activation of IRF-3 and NF-κB in DCs and macrophages (19). In contrast, no protective role of TLR4 has been reported in cases of WNV infection. TLRs interact with the viral replication products and transmit signals through a series of adaptor molecules, resulting in the production of pro-inflammatory cytokines (20). Both toll-like receptor 7 (TLR7) and TLR8 recognize ssRNA, and the recognition may have species specificity. Murine TLR7 and human TLR8 recognize SS GU-rich RNA as a natural ligand (21). Stimulation of TLR7 or TLR8 results in the subsequent activation of TRIF and MyD88-dependent signaling pathways that, in turn, induce the expression of pro-inflammatory cytokines, chemokine’s, and type 1 IFNs.

The role of TLRs in flaviviral infections remains elusive because of conflicting data. Previous studies have indicated that TLR7 recognizes WNV and is capable of mediating innate immune responses. However, an *in vivo* study reported that ablation of TLR7 in encephalitis due to JEV did not have significant progression effect (19). Furthermore, there was no change in susceptibility to JEV infection in TLR7-deficient mice. In addition, a recent study conducted by Nazmi and co-workers reported increased survival in mice treated with a TLR7 agonist compared with wild-type mice due to the use of lethal dose of JEV (GP78 strain, 3 × 10^5^ pfu/mouse) through subcutaneous route to induce infection. The reasons for the discrepancy in these results are unclear. Therefore, we aimed to determine whether the TLR7 signaling pathway modulates innate immune responses and viral pathogenesis following JEV infection in TLR7-deficient mice.

## Materials and Methods

### Mice

TLR7^−/−^ mice were provided as a courtesy by Dr. Ling Zhao, Huazhong Agricultural University. C57BL/6 mice were provided by Provincial Center for Disease prevention and control Hubei; Wuhan, China, and were transferred to the experimental station under the guidance of the general experimental animal transfer protocol. All mice were kept under specific-pathogen-free conditions (SPF). Experiments were performed on 6- to 8-week-old animals. Mice were separately housed according to their age and sex and grouped for identification and experimentation under identical conditions.

### Virus and Cells

The P3 strain of JEV generated from a Beijing isolate was used for both *in vitro* and *in vivo* studies. Inoculum of 15 μl of 5 × 10^4^ PFU of JEV-P3 virus was intracerebrally injected into the brain of 1-day-old suckling mice for the purpose of propagation (22). The morbid mice were euthanized and the brains were dissected. The brain tissue was homogenized to make 10% suspension (weight/volume) in Dulbecco Modified Eagle Medium. Then the prepared homogenate was centrifuged to remove cellular debris. The supernatant was separated and stored at −80°C. The virus titration was done in baby hamster kidney fibroblast cell line following the previously prescribed protocol (22).

### Bone Marrow Preparation

Mice were kept under SPF conditions in our own facility following the instructions of laboratory protocols. All of the procedures were performed under a safety cabin. Femurs and tibiae of TLR7^−/−^ and C57BL/6 mice were dissected, and the bones were kept free from muscle by rubbing with a tissue cleaner. For disinfection, the bones were placed in 70% ethanol up to 5 min and then washing was done with phosphate buffer saline (PBS). Disinfected bone ends were cut using autoclaved scissors, and the bone marrow was extracted into sterilized petri plates using PBS with the help of a syringe having a 0.45-mm diameter needle. The isolated material was pipetted to breakdown clumping and was then filtered into 50-ml tubes (Corning Centristar™) by a nylon cell separator with 0.40-µm pores (Falcon^®^, Corning, NY, USA). The filtrate was centrifuged at 12,000 rpm for 5 min. After one wash, the bone marrow cells were resuspended in RPMI 1640, USA culture media. Approximately 3.6 × 10^6^ leukocytes were obtained per femur or tibia after washing.

### Bone Marrow Cell Culture with GM-CSF

Introduction of GM-CSF into bone marrow-derived dendritic cells (bmDCs) was conducted as previously described (23, 24). Isolated bone marrow cells were plated in 12-well cell culture plates (LabServ^®^, Fisher Scientific, China) at a density of 2 × 10^6^ cells/ml in DC media [RPMI 1640 supplemented with 10% FBS (Gibco, Grand Island, NY, USA), 100 µg/ml streptomycin, 100 Ul/ml penicillin, and 10 ng/ml of rmGM-CSF and IL-4]. The incubation of these plates was performed at 37°C along with 5% CO_2_ in the incubator. Every 2 days post-culture, half of the supernatant media was exchanged with fresh DC culture media. On day 11, bmDCs were harvested for further processing.

### JEV-P3 Infection of bmDCs

The JEV-P3 virus stock was diluted and prepared prior to infecting the bmDCs. The cells were infected with a prepared JEV-P3 virus at 1 MOI. Plates of infected bmDCs were incubated at 37°C with 5% CO_2_. The samples for RNA extraction and flow cytometry were taken at 12, 24, 48, and 72 h post-infection. The supernatant was removed before sampling, and the cells were placed in Trizol^®^ Reagent (Invitrogen™, New Zealand) prior to storage at −80°C until further use for RNA extraction.

### JEV-P3 Inoculums in Mice

Total 25 of 6- to 8-week-old TLR7^−/−^ mice and 25 of C57BL/6 mice were allocated separately for animal experimentation under SPF conditions following the standard protocol. Mice were intravenously inoculated with JEV-P3 except for the control mice (*n* = 5) if needed. The dose rate of JEV-P3 was 1 × 10^5^ pfu/mouse, which was diluted with PBS at a pH of 7.4 and a final volume of 100 µl. The control animals were injected with the same dose of PBS by the same route. The animals were monitored at 2-day intervals for any clinical symptoms, mortality, and measurement of body weight. Data were recorded from the beginning until the end of the experiment. Some mice were sacrificed under sterile conditions on the day that they exhibited clinical symptoms, and tissue (brain and spleen) was collected from both TLR7^−/−^ and C57BL/6 mice. The dissected samples were preserved at −80°C until for further molecular analysis.

### Quantitative Real-time PCR to Detect Viral Load and Cytokine Expression

The tissue culture samples for RNA extraction were retrieved from −80°C storage and thawed for further processing. The viral RNA was extracted according to the given protocol (25). The extracted RNA was introduced for reverse transcription using a ReverTra Ace-α kit (Toyobo, Japan) in accordance with manufacturer’s directions. Quantitative reverse-transcription polymerase chain reaction (qRT-PCR) was accomplished by SYBR Green Real-Time PCR Master Mix (Takara, Japan) in accordance with manufacturer’s directions. The mRNA expression levels were normalized relative to β-actin. Data were calculated as the fold difference in the treatment compared with the control groups. Viral cDNA was detected using the JEV C gene specific forward and reverse primers.

### Flow Cytometry

Cell arrangement and flow analysis were conducted as described earlier (26). Rat anti-mouse monoclonal antibodies (eBioscience) were used for incubation of cells; fluorescence in isothiocyanate (FITC)-conjugated CD3, FITC- or phycoerythrin (PE)-conjugated CD4, antigen-presenting cell (APC)-conjugated CD8, FITC-conjugated CD11b, PE- or PE-Cy7-conjugated CD11c, FITC-conjugated CD19, APC-conjugated CD25, PE-Vio770-conjugated CD45R, PE-conjugated CD80, APC-conjugated CD86, APC-conjugated CD185 (CXCR5), PE-conjugated CD278 (ICOS), Percp-Cy5.5-conjugated CD279 (PD-1), FITC-conjugated PDCA-1, and/or APC-conjugated Gr-1 for 30 min at 4°C; after that it was washed using cold PBS before the flow analysis. Isotype-matched rat anti-mouse IgG antibodies (eBioscience) served as a negative control. Stained cells were analyzed on a FACS Calibur (BD) flow cytometry machine, and further calculations were performed with Graph pad software.

### Immunohistochemistry

The brain samples were collected from TLR7-deficient mice and C57BL/6 mice at the day 5 after JEV infection to determine the expression of TLR8 by using the immunohistochemical staining. In immunofluorescence, sections were incubated with primary rabbit anti-TLR8 pAb (1:50; abcam, China), followed by incubation with secondary antibody anti-rabbit IgG conjugated with Alexa Fluor 546. The same sections were then incubated with DAPI for nuclear counterstaining. Sections were imaged by using a fluorescence microscope (Olympus). Each group consists of (*N* = 3) mouse. The results were analysis after three independent experiments. TLR8 expression was analyzed through 10 fields of view (Figure 4C). The data were obtained and processed using Adobe Photoshop CS5 software (Adobe Systems, Pasadena, CA, USA).

### Statistical Analysis

Data was expressed as the means ± SEs (SEM). One-way analysis of variance subsequent to Tukey’s *post hoc* tests and two-tailed Student’s *t*-test was used to calculate the differences among different groups. Graphs were plotted and analyzed using GraphPad Prism, version 5.0 (GraphPad Software, La Jolla, CA, USA) (27).

## Results

### JEV Infection Triggers TLR7 Upregulation

**Figure 1:**
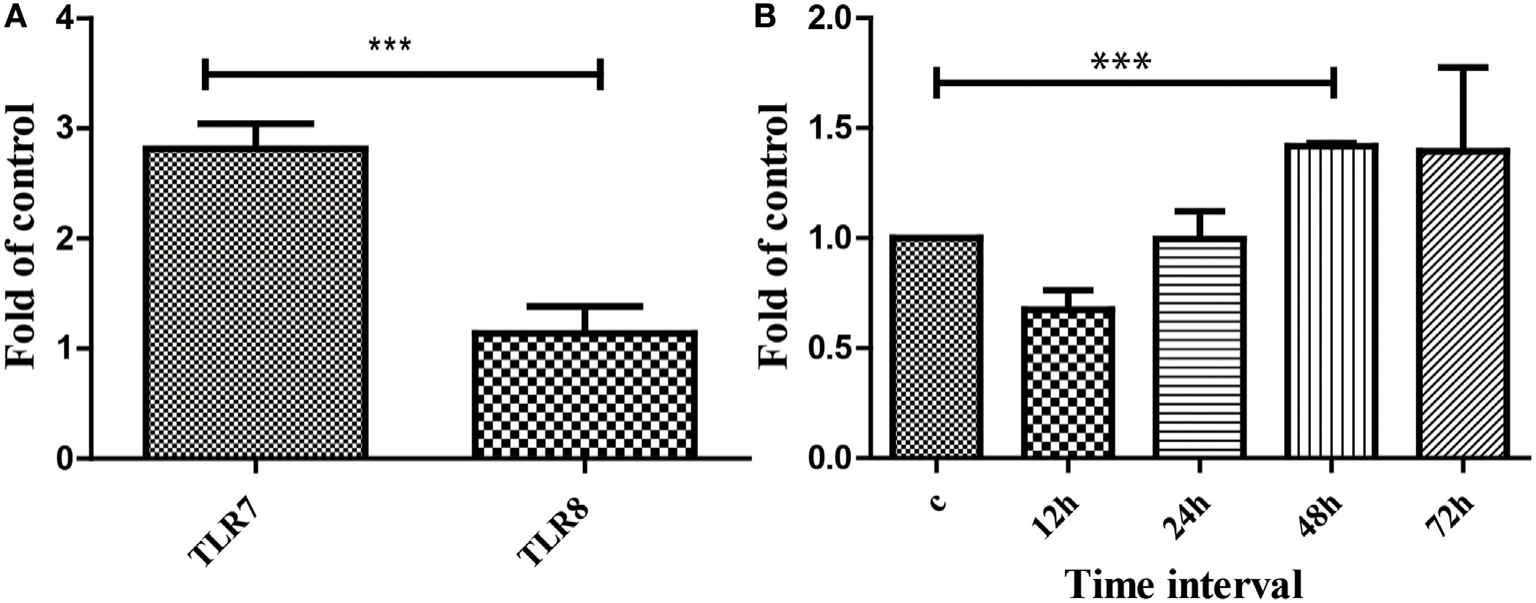
**Expression of toll-like receptors after Japanese encephalitis virus (JEV) infection**. **(A)** The expression of toll-like receptor 7 (TLR7), and TLR8 on the spleen after JEV infection in C57BL/6 mice. **(B)** The expression analysis of TLR7 on bone marrow-derived dendritic cells. C57BL/6 mice were infected with JEV, and the TLR7 expression rate was determined by quantitative reverse-transcription polymerase chain reaction at different time intervals (12, 24, 48, and 72 h). The real-time PCR results were analyzed by the ΔΔ*C*_t_ method and expressed as $2^{-\Delta C_{\text{t}}}$. The results are from three independent experiments. The data are presented as the mean ± SEM (***p < 0.001).

The JEV-P3 inoculated mice were monitored for the expression of two types of TLRs (TLR7 and TLR8) using qRT-PCR. Spleen cells from infected mice were used to check the expression of these TLRs. A threefold increase in TLR7 expression was observed, while TLR8 expression did not change significantly (Figure 1A). Based on these results, we selected TLR7 for further analysis. DCs represent the most important cell population in the initiation immune responses and serve as a bridge between innate and adaptive immunity. Furthermore, intracellular TLRs (TLR7 and TLR8) play a major role in the immune response against viral ssRNA through DCs. To further confirm the increase in TLR7 expression following JEV infection, bmDCs were introduced by JEV-P3 for infection on day 11 post-culture. TLR7 expression was determined by qRT-PCR at different time points (12, 24, 48, and 72 h) following infection. There was a 1.5-fold increases in TLR7 upregulation at 48 and 72 h. At 48 h post-infection, there was a significant rise in TLR7 expression (*p* < 0.001) (Figure 1B).

### TLR7 Deficiency Did Not Change Survivorship and Body Weight

**Figure 2:**
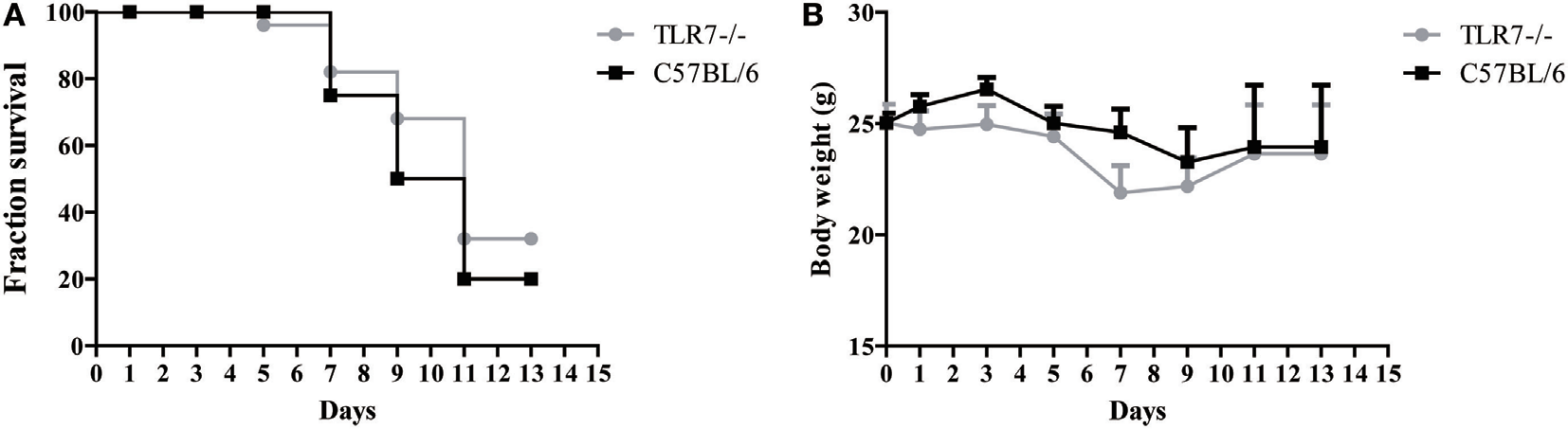
**Survivorship and body weight in toll-like receptor 7 (TLR7)-deficient mice post-Japanese encephalitis virus (JEV) infection**. Survival and body weight analysis in JEV-p3 infection, **(A)** 8-week-old age-matched C57BL/6 mice and TLR7^−/−^ mice were intravenously injected with 10^5^pfu of JEV-P3. Kalpan–Meier curves represent the survival distribution for mice over a period of 15 days, following challenge with *n* = 25 in each group. **(B)** The average of body weight change was monitored. Body weight was calculated every other day, beginning on day 0 until day 15. C57BL/6 infected group, *n* = 25; TLR7^−/−^ infected group *n* = 25. The *x* axis represents the time after infection (days), while the *y* axis represents the percent survival (%) and body weight. Each group consisted of *n* = 25 animals. The data are taken from duplicate sets of experiments.

Because the TLR7^−/−^ mouse strain was generated from the C57BL/6 strain, the latter were used as a control to compare JEV susceptibility. To determine the precise role of TLR7 in JEV pathogenesis, TLR7-deficient mice were inoculated with JEV (10^5^ pfu) intravenously. The infected mice were monitored at 2-day intervals for clinical symptoms, survivorship, and body weight. Less than a 10% increase in mortality was observed in TLR7-deficient mice compared with controls, which was not significant (Figure 2A). Similarly, the difference in body weight among TLR7-deficient and control mice groups was non-significant (Figure 2B). The above results indicate that genetic differences have no impact on the progression of neuroinflammation in JEV infection.

### Increased Viral Load in the Brain of TLR7-Deficient Mice

**Figure 3:**
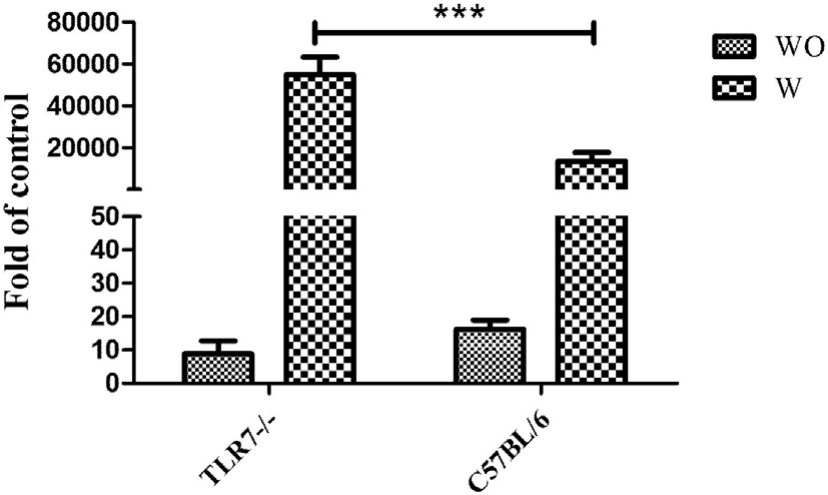
**Toll-like receptor 7 (TLR7) knock-out resulted in increased viral load in the brain following Japanese encephalitis virus (JEV)-infection**. TLR7^−/−^ mice were inoculated with JEV (10^5^ pfu), and the brain was collected after symptoms developed. Mice were divided to two groups according to the clinical signs. Once the clinical sign appeared, mouse has been sacrificed and samples have been collected as symptomatic groups (W) until 11 days of infection; and the left mice which did not show disease sign have been sacrificed at day 11 after infection and samples have been collected as asymptomatic (WO). The copy number of JEV-C genes in the brain was monitored with quantitative reverse-transcription polymerase chain reaction (qRT-PCR). The viral load was determined by qRT-PCR in the brain in the asymptomatic (WO) and symptomatic groups (W) of C57BL/6 and TLR7^−/−^ mice following JEV infection. The real-time PCR results were analyzed by the ΔΔ*C*_t_ method and expressed as $2^{-\Delta C_{\text{t}}}$. The results are from three independent experiments. The data are presented as the mean ± SEM (***p < 0.001).

To compare the viral load between C57BL/6 and TLR7^−/−^ mice, brain samples were assessed with qRT-PCR amplification of the viral C-gene. After inoculation of virus, some animal show clinical symptom from fourth or fifth days and some does not show clinical symptom in whole experimental process in both TLR7^−/−^ and C57BL/6 groups. Based on the clinical symptoms, mice were further sub-divided into two groups, asymptomatic (WO) and symptomatic group (W) for the C57BL/6 mice and TLR7^−/−^ mice. It consists of *n* ≥ 5 mice for each group. Viral load was significantly increasing in symptomatic TLR7^−/−^ mice compared with C57BL/6 mice (*p* < 0.0001), the difference between asymptomatic TLR7^−/−^ and C57BL/6 mice was non-significant (Figure 3). Therefore, the JEV burden was greater in the brain tissue of TLR7^−/−^ mice than C57BL/6 mice in the symptomatic group, indicating that TLR7 plays a role in JEV infection.

### Response of TLR8 in TLR7-Deficient Mice Post-JEV Infection

**Figure 4:**
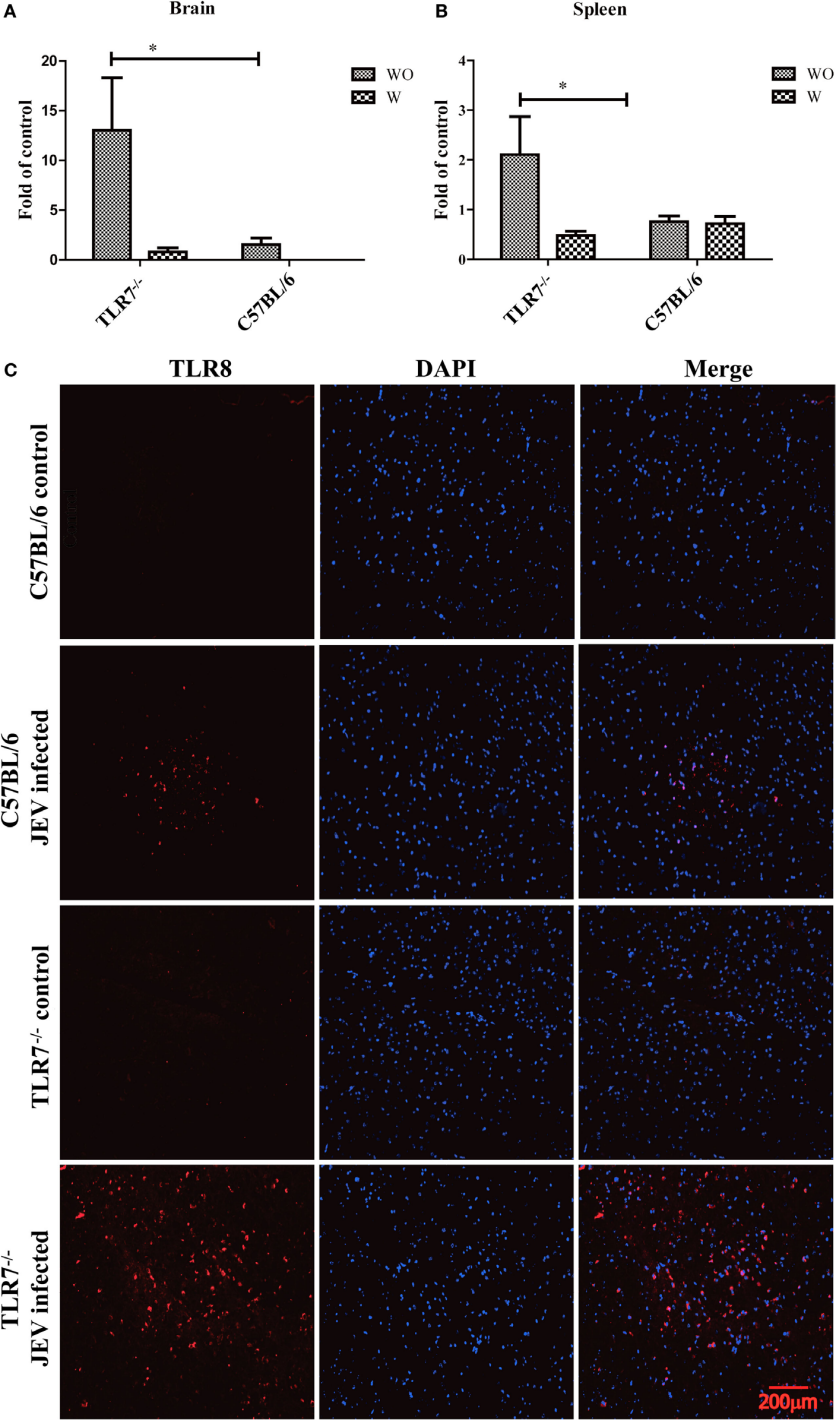
**Toll-like receptor 8 (TLR8) is upregulated in toll-like receptor 7 (TLR7)-deficient mice following Japanese encephalitis virus (JEV) infection**. **(A)** TLR8 expression in the asymptomatic (WO) and symptomatic groups (W) of brain samples was determined by quantitative reverse-transcription polymerase chain reaction (qRT-PCR) in TLR7^−/−^ mice and C57BL/6 mice after JEV infection. **(B)** The expression of TLR8 in spleen samples was determined in the asymptomatic (WO) and symptomatic groups (W) by qRT-PCR after JEV infection. **(C)** TLR8 expression was determined on brain by immunofluorescence staining in TLR7-deficient mice and C57BL/6 mice after JEV infection (red dot for TLR8). DAPI stains for nuclei (blue). The real-time PCR results were analyzed by the ΔΔ*C*_t_ method and expressed as $2^{-\Delta C_{\text{t}}}$. The results are taken from three independent experiments. The data are presented as the mean ± SEM (*p < 0.05).

Previous results showed that TLR7 may have an antiviral function in JEV infection. However, there were no significant differences in the survival rate. The innate immune system is highly sophisticated, and its pathways engage in considerable crosstalk. The question arose whether other TLRs played compensatory roles in the immune response to JEV in TLR7-deficient mice. To answer this question, we analyzed the expression of other TLRs (TLR3 and TLR8) in TLR7-deficient mice. Interestingly, increased expression of TLR8 was observed in both the spleen and brain of JEV-infected TLR7-deficient mice (*p* < 0.0001). More than a 10-fold increase in TLR8 was observed in brain tissue, while approximately a 2-fold increase was observed in the spleen of TLR7^−/−^ mice compared with C57BL/6 mice (Figures 4A,B). Based on previous results, the expression of TLR8 in brain was further determined through immunohistochemical staining. The increased expression of TLR8 was also observed in TLR7-deficient mice as compare to C57BL/6 mice following JEV infection; while the expression of TLR8 was not significantly increase in uninfected TLR7-deficient mice as compare to C57BL/6 mice group (Figure 4C).

### Response of Surface Stimulatory and Inhibitory Molecules in TLR7-Deficient Mice

**Figure 5:**
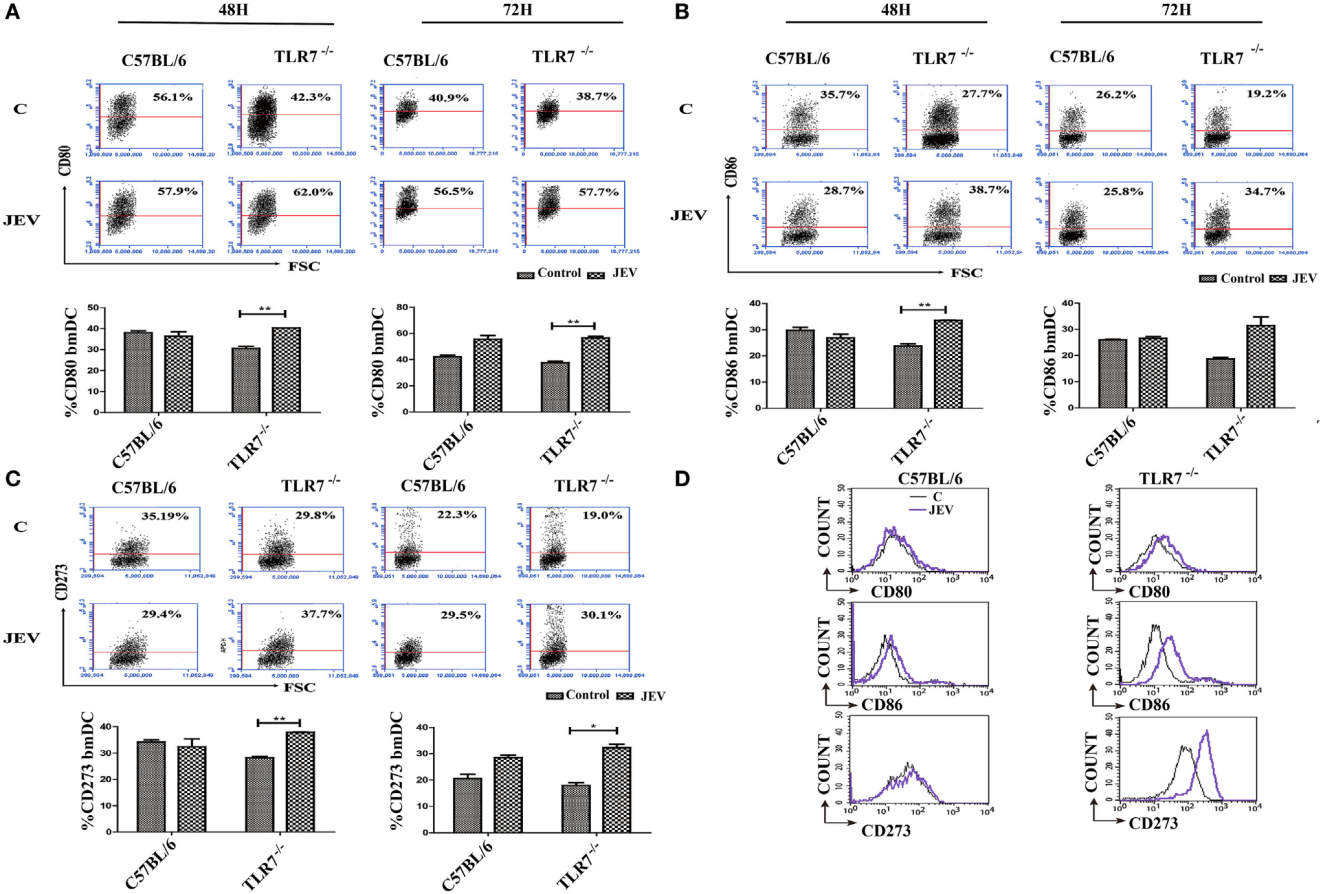
**Stimulatory and inhibitory surface molecule response on bone marrow-derived dendritic cell (bmDC) in toll-like receptor 7 (TLR7)-deficient mice**. **(A,B)** Co-stimulatory surface molecules of CD80 and CD86 were determined on bmDC in TLR7^−/−^ and C57BL/6 mice at 48 and 72 h after Japanese encephalitis virus (JEV) infection. **(C)** The inhibitory molecule of CD273 was determine on bmDC in TLR7^−/−^ and C57BL/6 mice at 48 and 72 h after JEV infection. **(D)** Co-stimulatory surface molecule and inhibitory molecules were determine on bmDC in TLR7^−/−^ and C57BL/6 mice at 48 h after JEV infection (black line: control, blue line: JEV). The flow cytometry data were analyzed with GraphPad software. The results were expressed after three independent experiments. The data are presented as the mean ± SEM (*p < 0.05; **p < 0.01).

The surface cell stimulator ligands CD80 and CD86 are key factors in the initiation and maintenance of immune responses. In TLR7^−/−^ and C57BL/6 mice, the expression of some surface cell stimulators, such as CD80 and CD86, and inhibitors, such as CD274 and CD273, in JEV-infected bmDCs was subsequently confirmed through flow cytometry. The results revealed that the expression of the surface stimulators CD80 and CD86 increased at 48 and 72 h after JEV infection in TLR7^−/−^ mice compared with the control group (*p* < 0.0102; *p* < 0.0083). There were no significant differences in JEV-infected C57BL/6 mice compared with control mice (Figures 5A,B). The expression of CD273 (cell surface inhibitor) was also significantly increased at 48 and 72 h (*p* < 0.0041). More than a 10% increase was observed in the JEV-infected group compared with the control group in TLR7^−/−^ mice at each time point (Figure 5C). Compared with control mice, bmDCs from TLR7^−/−^ mice showed a dramatical increase on both CD86 and CD273 (Figure 5D).

### Responses of T Subsets, DC, and pDC in TLR7-Deficient Mice

**Figure 6:**
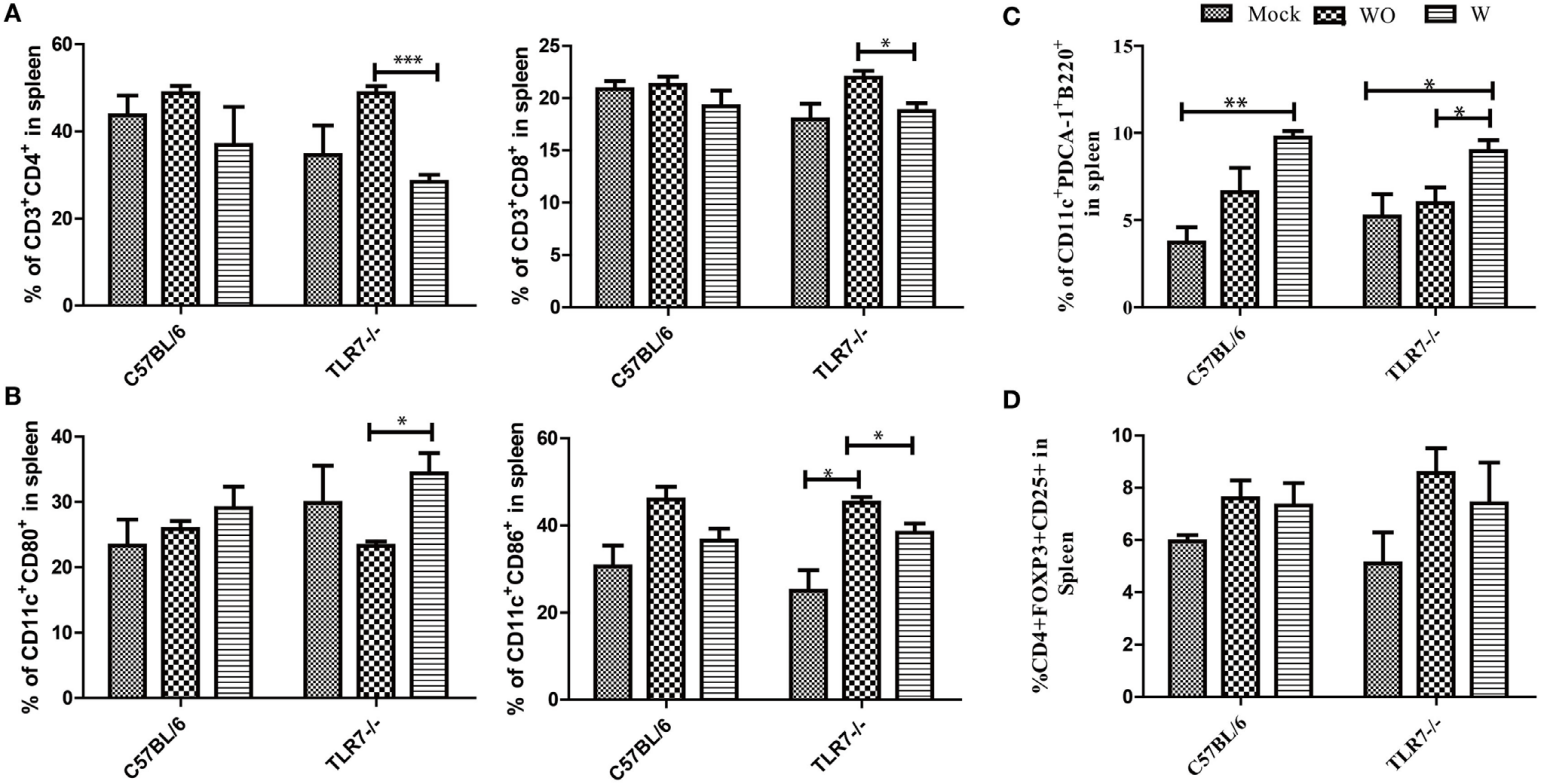
**Response of T subsets, dendritic cell (DC), and pDC in toll-like receptor 7 (TLR7)-deficient mice**. **(A)** The expression of T subset CD3^+^CD4^+^CD8^+^ was determined in different groups, such as the asymptomatic (WO), mock, and symptomatic groups (W) in TLR7^−/−^ mice and C57BL/6 mice. **(B)** Expression of the DC subset CD80^+^ CD11c^+^CD86^+^ was determined in the asymptomatic (WO), mock, and symptomatic groups (W) in TLR7^−/−^ mice and C57BL/6 mice after JEV infection. **(C)** The plasmacytoid DCs subset CD11c^+^mPDCA-1^+^CD45r/b220 was determine in the asymptomatic (WO), mock, and symptomatic groups (W) in TLR7^−/−^ mice and C57BL/6 mice post-JEV infection. **(D)** The ratio of the Treg subset CD4^+^CD25^+^Foxp3^+^ was determined in the asymptomatic (WO), mock, and symptomatic groups in TLR7^−/−^ mice and C57BL/6 mice post-JEV infection. Flow cytometry data were analyzed with GraphPad software. The results were expressed after three independent experiments. The data are presented as the mean ± SEM (*p < 0.05; **p < 0.01;***p < 0.001).

Dendritic cells are the most powerful APC. To make the transition from natural immunity to adaptive immunity, the changes in the T subsets, DC, and pDC in JEV infection, were investigated. The animals were divided into three groups, mock, asymptomatic (WO), and symptomatic (W), for both TLR7^−/−^ and C57BL/6 mice. T cell subsets, such as CD3^+^CD4^+^ and CD3^+^CD8^+^, were increased in the asymptomatic (WO) compared with the mock group. In TLR7^−/−^ mice, the cell number of the CD4^+^ T subset was increased in the asymptomatic group (WO) compared with the mock and symptomatic groups (W) (Figure 6A). The ratio of CD11c^+^CD80^+^ was increased significantly in the symptomatic group (W) compared with the mock and asymptomatic groups (WO) in TLR7^−/−^ mice. The ratio of CD11c^+^CD86^+^ was increased significantly in the asymptomatic group (WO) compared with the symptomatic (W) and mock groups in TLR7^−/−^ mice. In C57BL/6 mice, the ratio of CD11c^+^CD80^+^ and CD11c^+^ CD86^+^ were altered in the asymptomatic (WO) and symptomatic groups compared with the mock group. The number of CD11c^+^CD80^+^ and CD11c^+^CD86^+^ showed no significant increase in TLR7^−/−^ mice. In C57Bl/6 mice, the number of CD11c^+^CD80^+^ was not significantly increased, while the number of CD11c^+^CD86^+^ was significantly increased in the asymptomatic group (WO) compared with symptomatic (W) and mock groups (Figure 6B). The ratio of CD11c^+^PDCA^+^B220^+^ was increased in the asymptomatic group (WO) compared with the mock and symptomatic groups (WO) in both TLR7^−/−^ and C57BL/6 mice. The number of CD11c^+^PDCA^+^B220^+^ was significantly increased in the asymptomatic group (WO) compared with the symptomatic (W) and mock groups only in C57BL/6 mice (Figure 6C). The ratio of the Treg subset of cells, such as CD4^+^CD25^+^ FOXP3^+^, changed in both the symptomatic (W) and asymptomatic groups (WO) compared with the mock group in C57BL/6 mice and TLR7^−/−^ mice (Figure 6D). The number of CD4^+^CD25^+^FOXP3^+^ was significantly increased in the asymptomatic group (WO) compared with the symptomatic (W) and mock groups in C57BL/6 mice, while the number of CD4^+^CD25^+^ FOXP3^+^ was not significantly increased in TLR7^−/−^ mice.

## Discussion

Toll-like receptors have been broadly accepted as the first line of defense in the host defense mechanism and serve to stimulate the innate immune response and trigger the initiation of the adaptive immune response (28). Although the RIG-1and MDA5 in flaviviruses also initiate the type 1 IFN innate response in innate immunity (21, 29), the competency of TLRs as front line activators of innate immunity has not been investigated in the inter- and intracellular space. To our knowledge, the role of TLRs in the defensive mechanism against the progression of JE disease has not been studied. We observed that TLR7 was upregulated in spleen samples in C57BL/6 mice compared to other TLRs. The expression level of TLR8 was also significantly increased in the spleen and brain tissue of TLR7-deficient mice compared with C57BL/6 mice post-JEV infection. The viral load was significantly increased in the brain tissue of TLR7-deficient mice compared with C57BL/6 mice post-JEV infection. JEV infection activated cells involved in innate immunity, such as DC and pDC, as well as cells that participate in adaptive immunity, such as CD4^+^ and CD8^+^ T cells, in TLR7-deficient mice.

A mouse model of WNV infection was used to study the role of TLR7 in triggering immunity (30). Previously, TLR7 was considered to be an immediate and principal defense element against ssRNA viral infection prior to viral replication in the host after entry (21). In a previous study, TLR7 was a highly efficient component of the JEV defense system, identifying viral ssRNA just after entry and before viral replication (31). In current study, we determined the expression levels of TLR7 and TLR8 signaling pathways on the spleen in C57BL/6 mice. We found that only the TLR7 signaling pathway was upregulated following intravenous JEV infection in C57BL/6 mice.

In a previous study, there was no difference in the survival rate between TLR7-deficient mice and wild-type controls after WNV infection (32). Moreover, compared to JEV-infected wild-type mice, the viral load was also significantly increased, and non-significant difference was perceived in survivorship and body weight in TLR7-deficient mice (31). In the current investigation, we found an increased viral load in the brain of TLR7^−/−^ mice, although there was non-significant difference in survivorship and body weight after JEV infection between TLR7-deficient mice and C57BL/6 mice. The inconsistency of survivorship with previous report may be because of different viral strains, infection doses, and routes. In the previous study, the authors used lethal dose of JEV (GP78 strain, 3 × 10^5^ pfu/mouse) through subcutaneous route to induce infection (31). In another study the authors also used lethal dose of JEV (Beijing-1 strain, 1.4 × 10^7^ and 2.8 × 10^7^ pfu/mouse) through intraperitoneally route to induce infection (19). But in the present study, we used sub-lethal dose of JEV (P3 strain, 1 × 10^5^ pfu/mouse) by intravenous route to induce infection in mice. Different viral strains have different virulence. Moreover, we used the mice with same genetic background in which individual’s immunological differences may also exist, which provoked us to divide the mice into different groups according to their symptoms. Furthermore, there was less than a 10% increase in susceptibility to JEV infection in TLR7^−/−^ mice, suggesting that the TLR7 response is not a main contributor to immunity following JEV infection. Surprisingly, we found that TLR8 was upregulated in TLR7-deficient mice compare with C57BL/6 mice post-JEV infection. This finding implies that the similarity in the survival rate and body weight between TLR7^−/−^ mice and C57BL/6 mice might due to the upregulation of TLR8. TLR8 may have a compensatory function in regulating the immune response to JEV infection in TLR7-deficient mice. TLR7 and TLR8 are phylogenetically very similar. Even the species-specific recognition has been reported that both human TLR8 and murine TLR7 use single-stranded RNA as natural ligand (21). However, human TLR7 and TLR8 can activate independently the recognition of the same antiviral compound imidazoquinoline R-848 (33). There is possibility that the intermediates of viral replication stimulate TLR8 in TLR7-deficient mice. Based on the former studies, the current investigation helps to better understand the TLR7/8 function in mice. TLR7 expression varies depending on the cell type and may be compensatory to TLR8 expression, which is important for myeloid cells regulation. TLR8 may exert a regulatory influence on the immune response by controlling TLR7 expression. In the absence of TLR8, the expression of TLR7 was upregulated in DCs and highly responded to TLR7 ligands along with stronger NF-κB activation (34). The surface cell stimulator ligands CD80 and CD86 play a vital part in the maintenance and initiation of the immune response against viral infections (35). In a previous study, we observed that stimulatory surface molecules were upregulated and pro-inflammatory cytokines were induced in C57BL/6 mice after infection (36). In this study, we also found that the expression of the surface stimulators CD80 and CD86 increased at 48 and 72 h after JEV infection in TLR7^−/−^ mice compared with control mice. Furthermore, the expression of CD273 (surface inhibitor cell) also significantly increased at 48 and 72 h in TLR7-deficient mice compared with control mice after JEV infection.

Type 1 IFN acts as bridge between innate and adaptive immunity (37, 38) by enhancing antigen-specific CD8^+^T cells (39) and CD4^+^T cells (40) and activating NK cells (41). In the previous study, T subsets were altered (19). We also investigated the change in the T subsets, DC response, and pDC response in JEV infection. We found an increase in the T subsets CD3^+^ CD4^+^ and CD3^+^ CD8^+^ in the JEV-infected group compared with the mock group in TLR7-deficient mice. While in C57BL/6 mice, the ratio of T subsets were not significantly increased in JEV infected group as compare to mock group. Thus, TLR7 molecules act as central mediators among adaptive and innate immunity in JEV infection. In TLR7^−/−^ mice, JEV infection induced higher levels of pDC and improved the responses of JEV-specific CD4^+^ and CD8^+^ T cells involved in viral clearance during the early and late phases of infection, respectively.

The DC subset and pDC subset of cells performs a key part in viral clearance following JEV infection (19). The pDC plays an important role in providing type 1 IFN in response to ssRNA virus infection (42). In the previous study, the ratio of the DC and pDCs subsets was increased in TLR4^−/−^ JEV-infected mice compared with wild-type mice (43). In our study, the expression of pDCs was also increased in TLR7-deficient mice in the JEV-infected group compared with the mock group, which highlighted that TLR7 is important for the regulation of the pDC function in JEV infection. The ratio of the DC subset of cells, CD11c^+^ CD80^+^ and CD11c^+^ CD86^+^, was also significantly increased in TLR7^−/−^ mice post-JEV infection compared with control mice. These results suggest that TLR7 plays a more important role in the regulation of DC subsets of cells in JEV infection. The role of CD4^+^CD25^+^Foxp3^+^ in acute viral disease is still controversial (44, 45). Moreover, CD4^+^CD25^+^Foxp3^+^Treg cells contribute to the adaptive and innate immune responses during acute viral infection (43). In a previous study, the number and frequency of CD4^+^CD25^+^Foxp3^+^ were enhanced in TLR3^−/−^ mice post-JEV infection (19). In the present study, TLR7 deficiency led to an increase in the ratio of the Treg subset of cells, CD4^+^CD25^+^Foxp3^+^, response in the JEV infected group compared with the control group.

Our *in vitro* and *in vivo* results suggest that TLR7 is comparatively superior to the other TLRs especially TLR8 and plays a very important role in the regulation of myeloid cells and the immune response against JEV infection. In TLR7-deficient mice, TLR8 was upregulated and may also regulate the immune response and increase the survival rate following JEV infection. Future studies should investigate whether TLR8 is interlinked with TLR7 in the regulation of the immune response against JEV infection.

## Conclusion

TLR8 is upregulated in TLR7^−/−^ mice. Moreover, there is no significant difference in survival between TLR7-deficient and C57BL/6 mice. This may be because, in TLR7^−/−^ mice, the TLR8 signaling pathway is involved in the induction of immunity. An increased viral load in the brain was also observed in TLR7-deficient mice. However, future research is needed to explore the underlying mechanism. It appears that the depletion of one TLR may be compensated for by another TLR.

## Ethical Statement

All animal experiments were performed with the approval of the Animal Ethics Committee (protocol No. Hzaumo-2015-018), College of Veterinary Medicine, Huazhong Agriculture University Hubei, China.

## Author Contributions

Experiment design: MC, MA, KeW, XL, and ZF; performance of experiment: MA, WQ, NZ, CW, and KunlunW; data analysis: MA, LZ, and MC; paper writing: MA, MC, and ZF.

## Conflict of Interest Statement

The authors declare that the research was conducted in the absence of any commercial or financial relationships that could be construed as a potential conflict of interest.
