# Supplementary material for: TLR7 Deficiency Leads to TLR8 Compensative Regulation of Immune Response against JEV in Mice
Source: Front Immunol. 2017 Feb 20;8:160. doi: 10.3389/fimmu.2017.00160 (PMC5316529; doi:10.3389/fimmu.2017.00160)
Supplement: Supplementary file 1 [file image_1.pdf]

Expression of TLR7 on brain in C57BL/6 mice post JEV infection

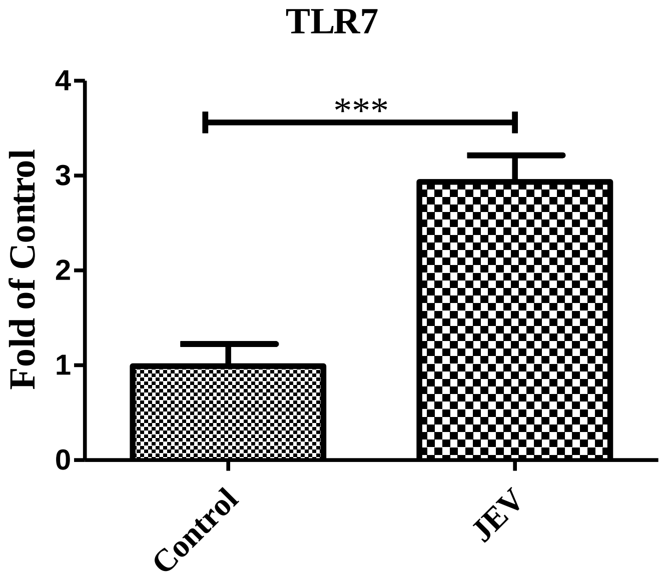

Expression of TLR7 on the brain in C57BL/6 mice post JEV infection. C57BL/6 mice were infected with JEV, and the TLR7 expression rate was determined by qRT-PCR. The real-time PCR results were analyzed by the ΔΔCt method and expressed as 2-ΔCt. The data are presented as the mean ± SEM. *** *P< 0.001.*
